# ChOLE-Based Stratification of Cholesteatoma Surgery: Predictive Value for Recurrence and Hearing Recovery

**DOI:** 10.3390/biomedicines13082040

**Published:** 2025-08-21

**Authors:** Yusuf Arslanhan, Ismail Aytac, Elif Baysal, Orhan Tunc, Berkay Guzel, Erhan Ciftel

**Affiliations:** 1 Department of Otorhinolaryngology, Viransehir State Hospital, 63700 Şanlıurfa, Turkey; 2 Department of Otorhinolaryngology, School of Medicine, Gaziantep University, 27310 Gaziantep, Turkey; dr.iaytac@gmail.com (I.A.); baysalelif@yahoo.com (E.B.); orhantip@hotmail.com (O.T.); jettjackson@gmail.com (E.C.); 3 Department of Otorhinolaryngology, University of Health Sciences, Basaksehir Cam and Sakura City Hospital, 34480 Istanbul, Turkey; drberkayguzel@gmail.com

**Keywords:** cholesteatoma, ChOLE classification, tympanomastoidectomy, chronic otitis media, hearing outcomes, ossicular chain, recurrence

## Abstract

**Objectives:** This study aimed to evaluate the clinical and hearing outcomes of patients with cholesteatomatous chronic otitis media using the ChOLE classification system and to assess its utility in predicting recurrence, guiding surgical approach, and anticipating hearing recovery. **Materials and Methods:** This retrospective study included 130 patients (141 ears) who underwent surgery for cholesteatoma between 2011 and 2020. Data were collected from surgical notes, imaging studies, and audiological evaluations. Patients were classified according to the ChOLE criteria, which incorporate cholesteatoma extension (Ch), ossicular chain status (O), and life-threatening complications (L). Surgical procedures and functional outcomes were compared across different stages and classification groups. Hearing outcomes were assessed in the early (3rd month) and late (2nd year) postoperative period. **Results:** Among the 130 patients (141 ears) evaluated, Stage II was the most common ChOLE stage (74.4%), followed by Stage I (17.7%) and Stage III (7.8%). The most frequently observed cholesteatoma extent was Ch3. A statistically significant association was found between surgical technique and ChOLE stage (*p* = 0.001): canal wall-down (CWD) tympanomastoidectomy was performed in 91% of Stage III cases and 84% of Stage II cases, whereas 76% of Stage I cases underwent canal wall-up (CWU) procedures. The overall recurrence rate was 29.5% in the CWU group and 16.4% in the CWD group, although this difference did not reach statistical significance (*p* = 0.792). However, ossicular chain status (O) showed a strong association with both early and late hearing outcomes. At 2 years, conductive hearing success was achieved in 90.9% of O0 patients, compared to 14% of O3b and 0% of O4 patients (*p* = 0.001). With regard to cholesteatoma extent (Ch), a statistically significant correlation was observed with early air–bone gap (ABG) closure success (*p* = 0.008) and late air conduction thresholds (*p* = 0.015). Similarly, ChOLE stage was significantly associated with early conductive hearing success (*p* = 0.012) and late ABG closure (*p* = 0.015). Overall, long-term hearing outcomes were superior to early results. Hearing success increased from 19.1% to 24.8% for air conduction thresholds and from 23% to 31.2% for hearing gain when comparing early and late follow-up periods. **Conclusions:** The ChOLE classification proved useful in guiding surgical strategy and predicting functional outcomes in cholesteatoma surgery. Advanced stage and greater cholesteatoma extension were associated with more extensive surgical procedures and poorer hearing results. Long-term audiological follow-up provided more accurate insights into surgical success. The standardized ChOLE system facilitates consistent reporting and meaningful comparison across institutions and studies.

## 1. Introduction

Chronic otitis media (COM) with cholesteatoma is a progressive and potentially destructive middle ear disease that remains a significant clinical challenge in otologic practice. It is characterized by the presence of keratinizing squamous epithelium in the middle ear or mastoid cavity, which can lead to bone erosion, ossicular chain disruption, and, if untreated, serious intracranial complications, including meningitis, brain abscess, and sigmoid sinus thrombosis [1,2,3]. Due to its aggressive nature and potential morbidity, cholesteatoma almost always requires surgical management aimed primarily at eradication of the disease and prevention of recurrence. Secondary goals include the preservation or improvement of hearing function [4,5,6].

Given the complex pathophysiology and variable progression of cholesteatoma, there has been a long-standing need for a standardized classification system that allows for reliable comparisons of surgical techniques and outcomes across studies and institutions. Numerous classification systems have been proposed over the years, focusing on parameters such as disease extension, localization, etiology, and pathological features [6,7,8,9]. However, a universally accepted classification has yet to be established, and many systems lack the ability to comprehensively guide surgical decision-making or predict outcomes.

In an effort to address these limitations, Linder et al. introduced the ChOLE classification system in 2018 [10]. This multidimensional system evaluates four key domains: Ch (Cholesteatoma extension), O (Ossicular chain status after surgery), L (Life-threatening complications), and E (Eustachian tube function, assessed radiologically via mastoid pneumatization). Unlike earlier systems that focused solely on disease extent or location, the ChOLE classification incorporates both preoperative and intraoperative findings, enabling a more holistic assessment of disease severity.

Since its introduction, only a limited number of studies have assessed the clinical applicability and prognostic value of the ChOLE system in real-world settings [11,12,13,14]. Initial evidence suggests that ChOLE not only facilitates standardization in documentation and reporting but also serves as a useful tool in guiding surgical approach and predicting outcomes, particularly in terms of recurrence risk and functional hearing restoration.

The importance of disease classification in cholesteatoma extends beyond academic consistency; it directly affects surgical planning, patient counseling, and long-term follow-up. An effective classification system should reflect not only the anatomical burden of the disease but also its clinical consequences, including the risks of residual disease, recurrence, and audiological outcomes.

In this context, the present study aimed to evaluate the clinical utility and prognostic value of the ChOLE classification in a cohort of patients who underwent surgery for cholesteatoma. By analyzing surgical techniques, complication rates, and functional outcomes in relation to ChOLE parameters, this study sought to determine the classification system’s effectiveness in predicting recurrence, hearing recovery, and surgical strategy selection.

## 2. Materials and Methods

### 2.1. Study Design and Study Population

Ethical approval for the study was obtained from the Clinical Research Ethics Committee prior to its initiation (approval no: 2021/240; date: 18 August 2021). A total of 141 patients with chronic otitis media who underwent surgery at the Department of Otorhinolaryngology, Head and Neck Surgery, Gaziantep University Faculty of Medicine, between November 2011 and March 2020, were retrospectively evaluated.

Patient data were collected from surgical records, epicrisis reports, radiological imaging, and audiological assessments. Patients diagnosed with middle ear cholesteatoma based on preoperative, intraoperative, or postoperative findings—including imaging and histopathological confirmation—were included in the study. Cases not meeting the inclusion criteria were excluded.

**Figure 1:**
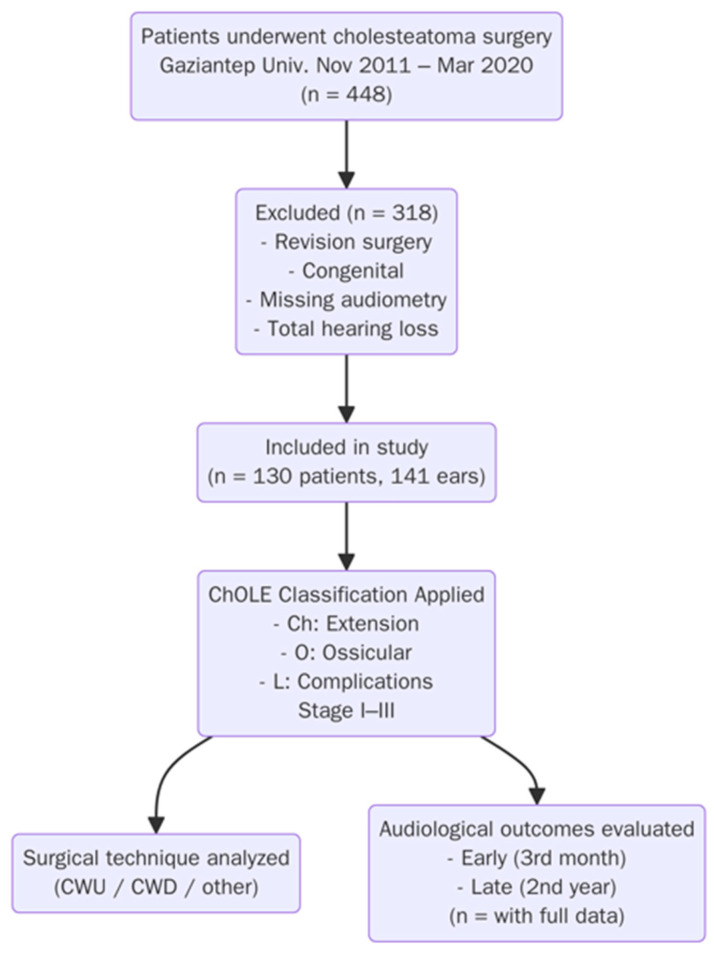
Flowchart of study.

Collected data included age, side of involvement (unilateral or bilateral), type of surgical procedure, duration of follow-up, presence of residual or recurrent disease, and adverse events. According to the ChOLE classification system [13,15], each patient was retrospectively evaluated for cholesteatoma extension (Ch), ossicular chain status (O), and the presence of life-threatening complications (L). Disease staging was performed using the official online ChOLE tool, classifying patients into Stages I–III. In cases where temporal bone computed tomography (CT) was not available, the extent of disease was recorded as “Ex” and excluded from staging-related analyses (Table 1, Figure 1).

### 2.2. Surgical Procedures

The choice of surgical technique was determined based on a combination of preoperative otomicroscopic examination, radiological imaging findings (temporal bone computed tomography), and intraoperative observations. Following the removal of the cholesteatoma—either through canal wall-down (open cavity), canal wall-up (intact canal wall), radical mastoidectomy, or atticotomy combined with tympanoplasty—reconstruction of the tympanic membrane was performed using temporalis fascia, conchal or tragal cartilage, or perichondrium, applied via the over–underlay technique. For ossicular chain reconstruction, the method varied depending on the extent and location of ossicular damage. Options included autologous ossicle repositioning, cartilage grafts, cortical bone grafts, or the use of titanium prostheses, specifically total ossicular replacement prosthesis (TORP) or partial ossicular replacement prosthesis (PORP). In cases where TORP or PORP were used, proper alignment with the oval window was ensured, typically maintaining a 90° angle, and contact between the prosthesis and a conchal cartilage graft was established to optimize sound transmission and prosthesis stability. All procedures were performed under an operating microscope, which allowed for adequate visualization of the middle ear structures. The decision between canal wall-up and canal wall-down techniques was primarily based on the extent of disease, anatomical considerations, and intraoperative findings. Transcanal endoscopic ear surgery (TEES) was not applied in this study.

### 2.3. Patient Follow-Up and Audiological Evaluation

All audiometric assessments were performed using a clinical audiometer (Interacoustics CA40, Interacoustics A/S, Middelfart, Denmark) and were interpreted according to the criteria established by the Japanese Society of Otology. Pure-tone averages (PTAs) were calculated for both air and bone conduction thresholds at 500, 1000, 2000, and 4000 Hz. Hearing gain was defined as the difference between postoperative air conduction and bone conduction thresholds. A successful functional outcome was defined by meeting at least one of the following criteria:

1. Postoperative air conduction threshold ≤ 30 dB.
2. Air–bone gap (ABG) ≤ 15 dB.
3. Hearing gain ≥ 15 dB.

### 2.4. Statistical Analysis

All statistical analyses were performed using IBM SPSS Statistics for Windows, version 27.0 (IBM Corp., Armonk, NY, USA). Continuous variables were expressed as the mean ± standard deviation (SD) or median (minimum–maximum) based on data distribution, while categorical variables were presented as frequencies and percentages. The Chi-square test was used to examine associations between categorical variables, including surgical technique and ChOLE parameters. For all analyses, a *p*-value < 0.05 was considered statistically significant.

## 3. Results

Of the 448 patients operated on for cholesteatoma, 130 met the inclusion criteria and were included in this study. Patients were excluded if they had undergone revision surgery in the same ear, had congenital cholesteatoma, lacked preoperative or early and late postoperative audiometry, or developed total postoperative hearing loss. A total of 141 ears were evaluated, as 11 patients had bilateral disease. The study population consisted of 74 males (52.5%) and 67 females (47.5%), with a mean age of 25.2 years (range: 4–73). Pediatric patients under the age of 18 accounted for 33% (*n* = 47). Surgeries were performed on the right ear in 48.2% (*n* = 68) and on the left ear in 51.8% (*n* = 73); 11 patients underwent bilateral surgery. The most common cholesteatoma extent was Ch3, and the most frequent ChOLE stage was Stage II. Regarding ossicular chain status, O1 was the most prevalent category, as most patients had partial ossicular erosion. The most frequently performed surgery was open cavity tympanomastoidectomy (CWD-TM) (66.6%), followed by intact canal tympanomastoidectomy (CWU-TM) and tympanoplasty. Surgically, 44 patients (31.2%) underwent CWU procedures: 20 (14.1%) had tympanoplasty alone, and 24 (17%) had intact canal TM. CWD procedures were performed in 97 patients (68.7%), including 94 open cavity mastoidectomies (66.6%), 2 modified radical mastoidectomies (1.4%), and 1 radical mastoidectomy (0.7%).

Distribution of operation type by cholesteatoma spread (ch) and stage in Chole classification is shown in Table 2. There was a statistically significant association between the type of surgery performed and both the extent (Ch) and stage of cholesteatoma (*p* = 0.001 for both comparisons). In early-stage disease (Stage 1), tympanoplasty was the most frequently performed procedure (60%), followed by intact canal wall tympanomastoidectomy (29.2%). As the stage progressed, more extensive surgical approaches were employed; notably, 84% of Stage 2 cases underwent open cavity surgery, and all Stage 3 patients were managed with open or modified radical mastoidectomy. Similarly, in patients with limited cholesteatoma spread (e.g., Ch1a), conservative procedures like tympanoplasty predominated, whereas open cavity techniques were favored in more advanced extensions such as Ch3 (Table 2).

Distribution of mastoidectomy method according to stage in Chole classification is shown in Table 3. There was a statistically significant correlation between the stage of cholesteatoma and the mastoidectomy method employed (*p* = 0.001). In Stage 1 patients, atticotomy was predominantly used (81.8%), reflecting the less extensive nature of early disease. As the stage progressed, more invasive techniques were favored: inside–out mastoidectomy and regular mastoidectomy were the most commonly performed procedures in Stage 2 (79.5% and 80%, respectively), while in Stage 3, regular mastoidectomy was performed in 11.1% of cases (Table 3).

Evaluation of early-term functional outcomes according to cholesteatoma expansion (ch) in Chole classification is shown in Table 4. There was a statistically significant association observed between cholesteatoma extent and air–bone gap (ABG) success (*p* = 0.008), indicating that early ABG outcomes vary with disease spread. Specifically, patients with limited disease (e.g., Ch1a and Ch1b) showed relatively higher rates of ABG success (25.0% and 17.9%, respectively), while more extensive involvement (e.g., Ch2b and Ch3) was associated with lower success rates. However, no statistically significant relationship was found between cholesteatoma extent and airway conductive hearing (*p* = 0.197) or hearing gain (*p* = 0.453) in the early postoperative period (Table 4).

Evaluation of late functional outcomes according to cholesteatoma expansion (ch) in Chole classification is shown in Table 5. There was a statistically significant correlation found between cholesteatoma extent and airway conductive hearing success (*p* = 0.015), indicating that long-term conductive hearing outcomes vary according to the severity of disease. Patients with limited cholesteatoma (e.g., Ch1a and Ch2b) showed higher success rates (25.7%) compared to those with extensive involvement (e.g., Ch3: 17.1%). While hearing gain and air–bone gap (ABG) success rates tended to decrease with increasing disease extent, these relationships did not reach statistical significance (*p* = 0.300 and *p* = 0.068, respectively) (Table 5).

Evaluation of early term functional outcomes according to ossicular chain (o) status in Chole classification is shown in Table 6. There was a statistically significant association between ossicular chain status and early postoperative outcomes in terms of airway conductive hearing (*p* = 0.015) and air–bone gap (ABG) closure (*p* = 0.017). Patients with an intact ossicular chain (O0) had the best outcomes, with a 25.9% success rate in both conductive hearing and ABG closure, and no gain failures. In contrast, more severely damaged ossicular statuses such as O3b and O4 showed markedly lower success rates. O3b patients, for instance, had high failure rates in both hearing gain (34.3%) and ABG (37.2%) despite some functional improvement. No successful outcomes were observed in the O4 group, indicating a strong negative impact of complete ossicular destruction on early hearing results (Table 6).

Evaluation of late-term functional outcomes according to ossicular chain(o) status in Chole classification is shown in Table 7. There was a statistically significant association between ossicular chain status and both 2-year postoperative conductive hearing success (*p* = 0.001) and air–bone gap (ABG) closure (*p* = 0.001). Patients with an intact ossicular chain (O0) and partial damage (O1) had the best long-term outcomes, with 28.6% success in both conductive hearing and ABG closure. In contrast, severely damaged ossicular statuses (O3b, O4, Ox) were associated with high failure rates. For example, O3b patients had a 44.3% failure rate in conductive hearing and a 42.6% failure rate in ABG closure. Notably, no successful hearing restoration was observed in the O4 group. Interestingly, hearing gain (*p* = 0.894) did not significantly differ across ossicular categories (Table 7).

Evaluation of early-term functional outcomes by stage in Chole classification is shown in Table 8. There was a statistically significant relationship between ChOLE stage and third-month postoperative conductive hearing success (*p* = 0.012). Patients with Stage 1 disease achieved the highest success rate in conductive hearing (33.3%), while no patients with Stage 3 disease demonstrated successful early hearing outcomes. Although differences in hearing gain and air–bone gap (ABG) closure were observed across stages, they did not reach statistical significance (*p* = 0.537 and *p* = 0.107, respectively) (Table 8).

Evaluation of late functional outcomes by stage in Chole classification is shown in Table 9. There was a statistically significant correlation between ChOLE stage and 2-year postoperative conductive hearing (*p* = 0.001) and air–bone gap (ABG) closure (*p* = 0.015). Patients with Stage 1 disease had significantly better outcomes, with 37.1% achieving successful conductive hearing and 32.5% achieving ABG closure. In contrast, Stage 2 patients exhibited higher failure rates across all metrics, particularly in ABG closure, where 80.2% were unsuccessful. Although hearing gain at 2 years showed a trend toward better outcomes in Stage 1, this difference was not statistically significant (*p* = 0.830) (Table 9).

## 4. Discussion

In this study, we aimed to evaluate the clinical utility of the ChOLE classification system in predicting surgical strategy and postoperative hearing outcomes in patients with chronic otitis media associated with cholesteatoma. The findings demonstrate a significant correlation between the ChOLE components—particularly cholesteatoma extension (Ch), ossicular chain status (O), and overall stage—and both the type of surgery performed and functional outcomes in early and late postoperative periods. In line with previous research, patients with more extensive disease or advanced ChOLE stages were more likely to have undergone open cavity or radical mastoidectomy, and their postoperative auditory results were significantly poorer compared to those in earlier stages. These results support the prognostic value of the ChOLE classification in clinical decision-making and underscore its potential as a standardized tool for inter-study and inter-center comparisons.

Although chronic cholesteatomatous otitis media (CCOM) was reported to be more prevalent in developing countries due to delayed access to healthcare services and limited resources, both congenital and acquired forms of cholesteatoma were recognized worldwide and posed significant clinical challenges regardless of geographic setting [10]. Congenital cholesteatomas typically originated from epithelial remnants within the temporal bone, whereas acquired cholesteatomas often developed as a consequence of chronic Eustachian tube dysfunction and tympanic membrane retraction. Given the potential of CCOM to progress silently and eventually lead to life-threatening intracranial complications such as meningitis, brain abscess, or sigmoid sinus thrombosis, early diagnosis followed by timely and appropriate surgical intervention was considered crucial in preventing morbidity and mortality [16]. In this context, the present study aimed to contribute to the existing literature by providing a comprehensive analysis of both long-term surgical outcomes and audiological recovery, using the ChOLE classification system—a multidimensional staging model that was first proposed by Linder et al. in 2017 [10]. This system incorporated key clinical and intraoperative findings and was designed to standardize disease assessment and facilitate comparisons across studies.

Unlike earlier classification systems that primarily focused on the anatomical extent of disease or histopathological features, the ChOLE classification provides a comprehensive and multidimensional framework that includes not only the extent of cholesteatoma involvement (Ch) but also the ossicular chain status (O) observed during surgery, the presence of life-threatening complications (L) such as intracranial extension, and the assessment of Eustachian tube function (E) based on mastoid pneumatization patterns. This holistic and integrative approach allows surgeons to make more informed intraoperative decisions and promotes consistency in disease staging, which in turn facilitates objective comparisons between different studies and treatment centers through its standardized, online-accessible platform. To the best of our knowledge, this study is among the first to systematically evaluate the long-term surgical and functional outcomes of cholesteatoma management using the ChOLE classification. From a demographic perspective, the distribution of variables such as patient gender, age, and laterality of surgery in our cohort is consistent with that reported in previous studies, suggesting the representativeness of our patient sample [17,18,19]. Furthermore, the distribution of ChOLE stages within our cohort—Stage I: 17.7%, Stage II: 74.4%, and Stage III: 7.8%—closely mirrors earlier publications, reinforcing the observation that Stage II is the most frequently encountered disease stage [17,20]. This pattern suggests that most patients presented for surgical intervention before the disease had progressed to its most advanced and destructive stages, possibly due to improved diagnostic awareness and earlier referrals in recent years.

We also analyzed the correlation between ChOLE stage and mastoidectomy technique, an area not thoroughly addressed in prior studies. We categorized mastoidectomy technique into atticotomy, inside–out, and regular mastoidectomy, and observed that as the disease stage increased, regular mastoidectomy was preferred. A statistically significant association was found between ChOLE stage and the type of mastoidectomy.

The selection of surgical approach was primarily influenced by the extent and stage of the cholesteatoma, a relationship that is clearly supported by our data. Similar to the findings reported by Hajare et al. [21], CWU techniques were generally preferred in patients with limited disease, particularly in early-stage cholesteatoma, due to their potential advantages in preserving anatomical structures and facilitating better postoperative quality of life. In contrast, CWD procedures were more frequently chosen in advanced-stage cases, where disease extension was greater and complete visualization of the middle ear and mastoid cavity was critical for achieving total eradication of the cholesteatoma. In our study, CWU surgery was performed in 76% of Stage I cases, reflecting a conservative surgical strategy in early disease. Conversely, 91% of Stage III patients underwent CWD procedures, indicating a shift toward a more aggressive surgical approach as disease severity increased. The clear preference for CWD techniques in advanced cases was likely attributed to their advantage in providing wider surgical exposure, thereby allowing for more effective removal of disease and reducing the risk of residual or recurrent cholesteatoma.

Tympanomastoidectomy, whether performed using the CWU or CWD technique, was recognized as the standard and definitive surgical intervention for the management of CCOM [22]. The CWU approach, which preserves the posterior canal wall, is often preferred in selected cases due to its potential aesthetic and anatomical advantages, including a more natural ear canal contour and easier postoperative care. However, this technique has been consistently associated with higher recurrence rates, likely due to limited surgical exposure and the potential for residual disease in hidden recesses of the middle ear and mastoid. Therefore, it is recommended that patients undergoing CWU be preoperatively informed about the increased likelihood of requiring revision surgery. Our study findings corroborate these observations: recurrence was identified in 29.5% of patients who underwent CWU procedures, whereas the recurrence rate in the CWD group was significantly lower, at 16.4%, consistent with previously published data [23,24]. These results support the notion that while CWU may offer cosmetic and structural benefits, CWD remains superior in minimizing disease recurrence, particularly in cases with extensive cholesteatoma involvement.

We also observed that long-term hearing outcomes were more favorable than early postoperative results. While no significant correlation was found between ChOLE stage and 3-month ABG values, a significant association emerged at the 2-year mark. For example, in Stage I, hearing success increased from 36% at 3 months to 52% at 2 years. This suggests that early assessments may underestimate true functional recovery, emphasizing the importance of long-term follow-up.

One of the most critical components of the ChOLE classification system is the ossicular chain status (O), which has a substantial influence on postoperative hearing outcomes. Bächinger et al. demonstrated a significant association between ossicular integrity and functional hearing recovery—particularly highlighting the contrast between patients with an intact ossicular chain (O0) and those with extensive ossicular destruction (e.g., O3) [17]. In our study, conductive hearing success rates improved over time in nearly all ossicular categories, with the exception of O4, where no patient achieved successful hearing restoration, either in the early or late postoperative period. At the 2-year follow-up, 90% of patients with O0 status demonstrated successful conductive hearing outcomes, compared to only 14% in the O3 group and 0% in the O4 group. These findings highlight a strong inverse relationship between the severity of ossicular damage and the likelihood of auditory recovery, underscoring the prognostic value of intraoperative ossicular assessment in surgical planning and patient counseling. However, we attributed the low predictive value of early hearing assessments to incomplete cavity healing, which may interfere with reliable audiometric evaluation. Therefore, late follow-up hearing testing is essential to accurately assess hearing improvement and detect potential recurrences.

In recent years, TEES has emerged as a valuable alternative or adjunct to microscopic techniques in the management of cholesteatoma. Endoscopes allow for improved visualization of hidden recesses, such as the sinus tympani and anterior epitympanum, thereby facilitating more complete disease removal. Some reports suggest that the integration of endoscopic techniques may reduce recurrence rates and influence prognostic factors. However, the ChOLE classification system was primarily developed and validated in the context of microscopic surgery, and its predictive utility in patients undergoing TEES remains to be fully established. Future studies incorporating endoscopic approaches are needed to evaluate how such techniques may complement or modify the prognostic value of the ChOLE classification.

### Limitations of Study

This study has some limitations. One-third of our study population consisted of pediatric patients (<18 years). Although pediatric cholesteatoma surgery may differ from adult cases in terms of disease behavior and prognosis, we did not perform a separate subgroup analysis due to statistical constraints. Previous studies, including those by Wang et al. [25] and Fermi et al. [26], have emphasized that the ChOLE staging system may have limited prognostic value in pediatric patients compared to adults. Similarly, Marchand et al. [13] reported that ChOLE has limitations in predicting outcomes in children. Therefore, age-related differences should be considered when interpreting our results. In addition, the majority of patients were classified as ChOLE Stage II, which limited the statistical power for meaningful comparisons involving Stage I and Stage III cases. The retrospective design and involvement of a multicentric surgical team may have introduced variability in both surgical technique and clinical documentation, potentially affecting the consistency of the data. Furthermore, although the ChOLE classification system has gained broader acceptance in recent years, long-term postoperative outcomes remain underreported in the existing literature, which made it challenging to conduct robust comparisons with previously published studies.

## 5. Conclusions

In conclusion, this study supports the prognostic utility of the ChOLE classification system in the surgical management of patients with cholesteatoma. Among the evaluated cases, Stage II disease and Ch3-type cholesteatoma extension were the most frequently encountered presentations. In line with disease severity, CWD techniques were more commonly performed in advanced-stage cases, as they offer superior intraoperative visibility, thereby facilitating complete disease eradication and reducing the risk of recurrence. Postoperative hearing outcomes were found to be significantly influenced by ossicular chain status, with the highest rates of auditory success observed in patients with intact ossicles (O0), and no hearing improvement seen in those with complete ossicular destruction (O4). Additionally, long-term audiological assessments—particularly those performed at the 2-year follow-up—proved to be more reliable and reflective of true surgical success compared to early postoperative measurements, which may have been affected by incomplete healing or middle ear status. Overall, the ChOLE classification provides a standardized and clinically practical framework that enables objective staging of cholesteatoma, guides surgical planning, and allows for meaningful comparisons of outcomes across different surgeons and institutions. Its integration into routine clinical practice may improve prognostic accuracy and contribute to more consistent reporting in future cholesteatoma research.

## Figures and Tables

**Table 1:** Overview of ChOLE classification system (adapted from Linder et al. [10]).

Component	Definition/Grading
**Ch (Cholesteatoma extension)**	Ch1: Limited to one site (e.g., attic, mesotympanum)
	Ch2: Two sites
	Ch3: Multiple sites, extensive disease
	Ch4: Petrous apex involvement
	Chx: Extension not evaluable
**O (Ossicular chain status)**	O0: Intact ossicular chain
	O1: Malleus present, partial erosion
	O2: Stapes present, malleus/ incus absent
	O3a: Stapes superstructure present
	O3b: Stapes superstructure absent
	O4: No ossicular remnants
	Ox: Not evaluable
**L (Life-threatening complications)**	L0: No complications
	L1: Presence of extracranial complications (e.g., labyrinthine fistula, facial palsy) L2: Intracranial complications (e.g., meningitis, abscess, sinus thrombosis)
**E (Eustachian tube function—mastoid pneumatization)**	E0: Well-pneumatized mastoid
	E1: Moderately pneumatized
	E2: Poorly pneumatized/sclerotic

**Table 2:** Distribution of operation type by cholesteatoma spread (Ch) and stage in Chole classification.

		Operation Type					
		Tympanoplasty	Intact Channel	Open Cavity	Modified Radical	Radical Cavity	
		N (%)	N (%)	N (%)	N (%)	N (%)	*p*
Chole	Ch1a	11 (55)	6 (25)	5 (5.3)	0 (0)	0 (0)	0.001 *
	Ch1b	4 (20)	3 (12.5)	2 (2.1)	0 (0)	0 (0)	
	Ch2a	2 (10)	3 (12.5)	24 (25.5)	0 (0)	0 (0)	
	Ch2b	2 (10)	7 (29.2)	23 (24.5)	0 (0)	1 (100)	
	Ch3	1 (5)	5 (20.8)	33 (35.1)	2 (100)	0 (0)	
	Ch4	0 (0)	0 (0)	2 (2.1)	0 (0)	0 (0)	
	Chx	0 (0)	0 (0)	5 (5.3)	0 (0)	0 (0)	
Stage	1	12 (60)	7 (29.2)	6 (6.4)	0 (0)	0 (0)	0.001 *
	2	8 (40)	16 (66.7)	79 (84)	1 (50)	1 (100)	
	3	0 (0)	1 (4.2)	9 (9.6)	1 (50)	0 (0)	

* Significance at *p* < 0.05, Chi-square test.

**Table 3:** Distribution of mastoidectomy method according to stage in Chole classification.

		Mastoidectomy Method			
		Atticotomy	Inside–Out	Regular	
		N (%)	N (%)	N (%)	*p*
Stage	1	9 (81.8)	7 (17.9)	8 (8.9)	0.001 *
	2	2 (18.2)	31 (79.5)	72 (80)	
	3	0 (0)	1 (2.6)	10 (11.1)	

* Significance at *p* < 0.05, Chi-square test.

**Table 4:** Evaluation of early-term functional outcomes according to cholesteatoma expansion (Ch) in Chole classification.

		Postoperative 3rd Month Airway Conductive Hearing			Postoperative 3rd Month Gain			Postoperative 3rd Month Air–Bone Path		
		Successful	Unsuccessful		Successful	Unsuccessful		Successful	Unsuccessful	
		N (%)	N (%)	*p*	N (%)	N (%)	*p*	N (%)	N (%)	*p*
Chole	Ch1a	8 (29.6)	14 (12.3)	0.197	5 (15.2)	17 (15.7)	0.453	7 (25.0)	15 (13.3)	0.008 *
	Ch1b	3 (11.1)	6 (5.3)		1 (3)	8 (7.4)		5 (17.9)	4 (3.5)	
	Ch2a	4 (14.8)	25 (21.9)		8 (24.2)	21 (19.4)		1 (3.6)	28 (24.8)	
	Ch2b	5 (18.5)	28 (24.6)		7 (21.2)	26 (24.1)		7 (25.0)	26 (23.0)	
	Ch3	7 (25.9)	34 (29.8)		12 (36.4)	29 (26.9)		8 (28.6)	33 (29.2)	
	Ch4	0 (0)	2 (1.8)		0 (0)	2 (1.9)		0 (0)	2 (1.8)	
	Chx	0 (0)	5 (4.4)		0 (0)	5 (4.6)		0 (0)	5 (4.4)	

* Significance at *p* < 0.05, Chi-square test.

**Table 5:** Evaluation of late functional outcomes according to cholesteatoma expansion (Ch) in Chole classification.

		Postoperative 2nd Year Airway Conductive Hearing			Postoperative 2nd Year Gain			Postoperative 2nd Year Air–Bone Path		
		Successful	Unsuccessful		Successful	Unsuccessful		Successful	Unsuccessful	
		N (%)	N (%)	*p*	N (%)	N (%)	*p*	N (%)	N (%)	*p*
Chole	Ch1a	9 (25.7)	13 (12.3)	0.015 *	7 (15.9)	15 (15.5)	0.300	9 (22.5)	14 (12.9)	0.068
	Ch1b	6 (17.1)	3 (2.8)		2 (4.5)	7 (7.2)		6 (15.0)	3 (3.0)	
	Ch2a	4 (11.4)	25 (23.6)		5 (11.4)	24 (24.7)		5 (12.5)	24 (23.8)	
	Ch2b	9 (25.7)	24 (22.6)		13 (29.5)	20 (20.6)		9 (22.5)	24 (23.8)	
	Ch3	6 (17.1)	35 (33)		16 (36.4)	25 (25.8)		9 (22.5)	32 (31.7)	
	Ch4	0 (0)	2 (1.9)		0 (0)	2 (2.1)		0 (0)	2 (1.0)	
	Chx	1 (2.9)	4 (3.8)		1 (2.3)	4 (4.1)		2 (5.0)	3 (3.0)	

* Significance at *p* < 0.05, Chi-square test.

**Table 6:** Evaluation of early-term functional outcomes according to ossicular chain (O) status in Chole classification.

		Postoperative 3rd Month Airway Conductive Hearing			Postoperative 3rd Month Gain			Postoperative 3rd Month Air–Bone Path		
		Successful	Unsuccessful		Successful	Unsuccessful		Successful	Unsuccessful	
		N (%)	N (%)	*p*	N (%)	N (%)	*p*	N (%)	N (%)	*p*
Ossicular Chain	O0	7 (25.9)	4 (3.5)	0.015 *	0 (0)	11 (10.2)	0.174	7 (25)	4 (3.5)	0.017 *
	O1	7 (25.9)	25 (21.9)		8 (24.2)	24 (22.2)		4 (14.3)	28 (24.8)	
	O2	3 (11.1)	15 (13.2)		4 (12.1)	14 (13)		3 (10.7)	15 (13.3)	
	O3a	4 (14.8)	14 (12.3)		4 (12.1)	14 (13)		3 (10.7)	15 (13.3)	
	O3b	5 (18.5)	48 (42.1)		16 (48.5)	37 (34.3)		11 (39.3)	42 (37.2)	
	O4	0 (0)	3 (2.6)		0 (0)	3 (2.8)		0 (0)	3 (2.7)	
	Ox	1 (3.7)	5 (4.4)		1 (3)	5 (4.6)		0 (0)	6 (5.3)	

* Significance at *p* < 0.05, Chi-square test.

**Table 7:** Evaluation of late-term functional outcomes according to ossicular chain (O) status in Chole classification.

		Postoperative 2nd Year Air Conductive Hearing			Postoperative 2nd Year Gain			Postoperative 2nd Year Air–Bone Path		
		Successful	Unsuccessful		Successful	Unsuccessful		Successful	Unsuccessful	
		N (%)	N (%)	*p*	N (%)	N (%)	*p*	N (%)	N (%)	*p*
Ossicular Chain	O0	10 (28.6)	1 (0.9)	0.001 *	3 (6.8)	8 (8.2)	0.894	10 (25)	11 (10.9)	0.001 *
	O1	10 (28.6)	22 (20.8)		10 (22.7)	22 (22.7)		9 (22.5)	23 (22.8)	
	O2	5 (14.3)	13 (12.3)		8 (18.2)	10 (10.3)		5 (12.5)	13 (12.9)	
	O3a	4 (11.4)	14 (13.2)		5 (11.4)	13 (13.4)		6 (15)	12 (11.9)	
	O3b	6 (17.1)	47 (44.3)		16 (36.4)	37 (38.1)		10 (25)	43 (42.6)	
	O4	0 (0)	3 (2.8)		1 (2.3)	2 (2.1)		0 (0)	3 (3)	
	Ox	0 (0)	6 (5.7)		1 (2.3)	5 (5.2)		0 (0)	6 (5.9)	

* Significance at *p* < 0.05, Chi-square test.

**Table 8:** Evaluation of early-term functional outcomes by stage in Chole classification.

		Audio 3 Months			Gain 3 Months			Air–Bone Path 3 Months		
		Successful	Unsuccessful		Successful	Unsuccessful		Successful	Unsuccessful	
		N (%)	N (%)	*p*	N (%)	N (%)	*p*	N (%)	N (%)	*p*
Stage	1	9 (33.3)	16 (14)	0.012 *	4 (12.1)	21 (19.4)	0.537	9 (32.1)	16 (14.2)	0.107
	2	18 (66.7)	87 (76.3)		27 (81.8)	78 (72.2)		17 (60.7)	88 (77.9)	
	3	0 (0)	11 (9.6)		2 (6.1)	9 (8.3)		2 (7.1)	9 (8)	

* Significance at *p* < 0.05, Chi-square test.

**Table 9:** Evaluation of late functional outcomes by stage in Chole classification.

		Audio 2 Years			Gain 2 Years			Air–Bone Path 2 Years		
		Successful	Unsuccessful		Successful	Unsuccessful		Successful	Unsuccessful	
		N (%)	N (%)	*p*	N (%)	N (%)	*p*	N (%)	N (%)	*p*
Stage	1	13 (37.1)	12 (11.3)	0.001 *	9 (20.5)	16 (16.5)	0.830	13 (32.5)	12 (11.9)	0.015 *
	2	22 (62.9)	83 (78.3)		32 (72.7)	73 (75.3)		24 (60)	81 (80.2)	
	3	0 (0)	11 (10.4)		3 (6.8)	8 (8.2)		3 (7.5)	8 (7.9)	

* Significance at *p* < 0.05, Chi-square test.

## Data Availability

Data are available upon request to the corresponding author.
